# The Role of Epigenetics in Neuroinflammatory-Driven Diseases

**DOI:** 10.3390/ijms232315218

**Published:** 2022-12-02

**Authors:** Sebastiano Giallongo, Lucia Longhitano, Simona Denaro, Simona D’Aprile, Filippo Torrisi, Enrico La Spina, Cesarina Giallongo, Giuliana Mannino, Debora Lo Furno, Agata Zappalà, Rosario Giuffrida, Rosalba Parenti, Giovanni Li Volti, Daniele Tibullo, Nunzio Vicario

**Affiliations:** 1 Department of Biomedical and Biotechnological Sciences, Section of Biochemistry, University of Catania, 95123 Catania, Italy; 2 Department of Biomedical and Biotechnological Sciences, Section of Physiology, University of Catania, 95123 Catania, Italy; 3 Department of Medical, Surgical Sciences and Advanced Technologies G.F. Ingrassia, University of Catania, 95123 Catania, Italy; 4 Department of Chemical, Biological, Pharmaceutical and Environmental Sciences, University of Messina, 98166 Messina, Italy

**Keywords:** neurodegeneration, epigenetics, chronic neuroinflammation, Alzheimer’s disease, amyotrophic lateral sclerosis, Parkinson’s disease, multiple sclerosis

## Abstract

Neurodegenerative disorders are characterized by the progressive loss of central and/or peripheral nervous system neurons. Within this context, neuroinflammation comes up as one of the main factors linked to neurodegeneration progression. In fact, neuroinflammation has been recognized as an outstanding factor for Alzheimer’s disease (AD), amyotrophic lateral sclerosis (ALS), Parkinson’s disease (PD), and multiple sclerosis (MS). Interestingly, neuroinflammatory diseases are characterized by dramatic changes in the epigenetic profile, which might provide novel prognostic and therapeutic factors towards neuroinflammatory treatment. Deep changes in DNA and histone methylation, along with histone acetylation and altered non-coding RNA expression, have been reported at the onset of inflammatory diseases. The aim of this work is to review the current knowledge on this field.

## 1. Introduction

Neurodegenerative disorders are defined by the progressive loss of the neuronal population of the central nervous system (CNS) and/or peripheral nervous system (PNS), eventually impairing pyramidal or extrapyramidal tracts and cognitive and behavioral abilities [1]. The progressive neurological impairment is triggered by accumulation of molecules, ultimately resulting in (i) proteotoxic stress, (ii) enhanced oxidative stress, (iii) increased cell death, and (iv) neuroinflammation [1]. The latter is referring to an inflammatory state affecting the CNS, following insults such as infections, trauma, ischemia, and toxins and resulting in release of neurotoxic mediators including interleukin-1beta (IL-1β), IL-6, IL-8, IL-33, tumor necrosis factor (TNF), chemokine (C-C motif) ligand 2 (CCL2), CCL5, and other molecules which are mainly secreted by astrocytes and microglia [2,3]. Upon a chronic exposure to these molecules, neuroinflammation becomes detrimental, eventually becoming a critical point for the onset of neurodegenerative disorders. Neuroinflammation has been highlighted as an outstanding factor for Alzheimer’s disease (AD), amyotrophic lateral sclerosis (ALS), Parkinson’s disease (PD), and multiple sclerosis (MS) progression. In this regard, the main histopathological feature characterizing neurodegenerative disorders is an aberrant protein conformation resulting in its abnormal intercellular communication, impaired cell differentiation, and neuroanatomical distribution [4,5,6,7,8,9,10,11,12,13,14,15,16,17]. Depending on the nature of the accumulated protein, neurodegenerative diseases have been defined as amyloidosis, tauopathies, α-synucleinopathies, and transactivation response DNA binding protein 43 (TDP-43) proteinopathies [1].

**Figure 1:**
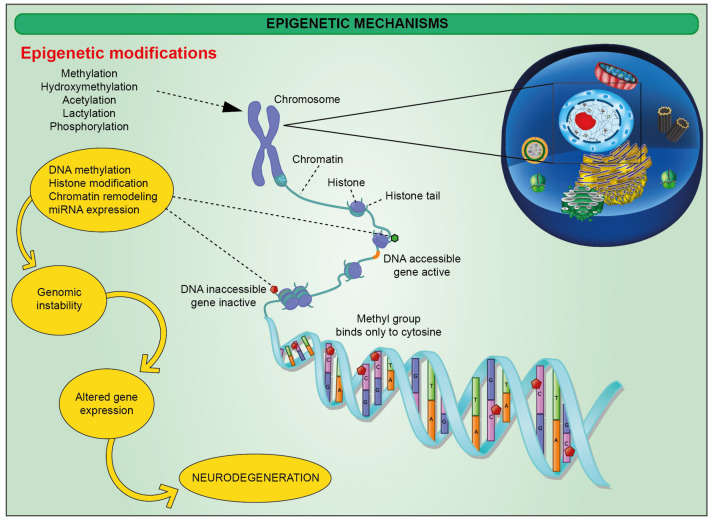
Schematic representation of epigenetic mechanisms affecting histones and DNA nucleobases resulting in modified DNA methylation status and genomic instability.

Diagnostic biomarkers are available only for neurodegenerative diseases linked to a causative genetic mutation [18,19,20,21]. Otherwise, the gold standard diagnostic strategy is based on the neuropathological evaluation at autopsy [22]. Moreover, pharmacological strategies treating the most common neurodegenerative diseases show limited therapeutic effectiveness, merely slowing down the disease progressions. To improve this outcome, a better characterization of molecular mechanisms behind neurodegeneration is needed. For this reason, the latest studies have also focused on the epigenetic profile characterizing these neuropathologies. Epigenetic changes include a plethora of modifications affecting gene expression without altering the DNA sequence, in turn determined by age, diet, stress, and disease state [23,24]. They include changes in chromatin architecture mediated either by chemical modifications affecting DNA nucleobases and histones (Figure 1) [25] or exchange of histone non-canonical isoforms on the nucleosome itself [23,26,27].

As a result, epigenetics drives chromatin from a loose, transcriptionally active euchromatin state towards a transcriptionally repressed heterochromatin and vice versa [26,27]. Not only epigenetics but also modifications affecting the chromatin 3D structure might be a determinant factor in this context. As it has been reported recently, elements of 3D architecture such as compartments, frequently interacting regions (FIREs), and loops might address the cell-type-specific gene regulation, providing hints on understanding the cellular etiology of brain diseases [28]. In this scenario, regulating gene expression in a tissue- and cell-specific way emerged as a critical level of control, eventually playing an important part also in cell differentiation, highly inducing the exploration of potential cell-based approaches [29,30,31,32,33,34,35,36]. While the classical epigenetic profile is usually propagated to the progeny, neuroepigenetics, referred to as epigenetic changes affecting CNS cell populations, involve modifications that cannot be inherited, since neurons do not divide [37]. For instance, DNA methylation plays a pivotal role in brain development, as DNA methyltransferases (DNMT1, DNMT3A, and DNMT3B) mediate memory acquisition and storage [38,39]. DNMT1 and DNMT3A deletion in mice exploits hippocampus-based learning and memory, eventually also blocking fear conditioning [38,40]. The latter promotes DNMT activity, ultimately suppressing the gene encoding the catalytic subunit of memory suppressor protein phosphatase 1 (PP1), in synergy with Reelin expression, encoding a plasticity-associated protein [38]. DNA is also affected by hydroxylation, a family of modifications involving the introduction of hydroxyl group(s) catalyzed by multiple types of hydroxylases [41]. Notably, the activity of this family of proteins relies on key nutrients, including Fe(II), 2-oxoglutarate (2OG), and ascorbate. Hydroxylation is gaining an important role in signaling, as it has been reported that 5-hydroxymethyl cytosine (5hmC) accumulation may recruit reader proteins towards specific genomic regions [42]. DNA demethylation is mediated by a family of proteins known as ten-eleven translocation protein (TET), including TET1, TET2, and TET3, which convert the DNMT product 5-methylcytosine in 5-hydroxymethylcytosine [43]. In mice, TET1 promotes demethylation of genes involved in memory formation and consolidation including brain-derived neurotrophic factor (BDNF), fibroblast growth factor (FGF), activity-regulated cytoskeletal-associated protein (ARC), early growth response protein 1 (EGR1), the transcription factor FOS, the scaffolding protein HOMER1, and nuclear receptor subfamily 4 group A member 2 (NR4A2). Indeed, TET1 depletion in mice models disrupts long-term contextual fear memory [44]. Furthermore, DNA methylation might be defined as an epigenetic clock, which has been modeled by Horvath [45], Hannum [46], and PhenoAge [47], and eventually strongly correlating with aging-related outcomes, physical functioning, and aging-related pathologies, including cognitive decline [47,48]. Of note, the cortical clock has been trained on samples from non-diseased individuals, using elastic net regression for the identification of relevant CpGs, showing a strong predictive value and outperforming Horvath’s clock in cortical bulk brain specimens [49,50].

Epigenetic modifications include histone modifications. Within this context, histone acetylation, mediated by histone acetyltransferases (HATs), covers a key role in autophagy and memory regulation. Of particular relevance is the evidence that histone acetylation in hippocampal neurons activates autophagy and inhibits NLRP3 inflammasome complex, which acts as an upstream activator of NF-κB signaling and induces NLRP3, cleaved caspase-1, and IL-1β [51,52]. Such a mechanism exerts protective effects on neuroinflammatory chronicization, and decreased levels of histone acetylation in aged neurons are likely triggering significant inflammatory responses via NLRP3 inflammasome complex [52]. The link between such a phenomenon and resident CNS immune cells activation still needs to be fully elucidated, even if it established the role of NLRP3 activation in AD and contributes to the progression of multiple inflammatory disorders [53,54,55]. It has been indeed shown that novel taste learning in mice triggers mitogen-activated protein kinase/extracellular signal-reduced kinases (MAPK/ERK) signaling, eventually leading to HATs activation [56]. As a result, there is an increase in the acetylation of genes located in the insular cortex, which is crucial for the formation of novel taste memories. In a contextual fear-conditioning experiment, an increase in mice of long-term potentiation at CA1 synapses in the hippocampus upon histone deacetylase inhibition has been reported [57]. Further studies supporting the outstanding role of histone acetylation in modulating synaptic plasticity used a mouse model where p25, a protein implicated in neurodegeneration, is conditionally expressed. Here, administration of HDAC inhibitors mitigated memory loss, even after neuronal impairment and brain atrophy [56]. Finally, human mutations impairing cAMP response element-binding protein (CREB)-binding protein (CBP; a HAT and crucial binding partner of CREB) are part of Rubenstein–Taybi syndrome and show an autosomal dominant disease characterized by severe learning disabilities [58]. Recent reports also included micro- and long non-coding RNA (miRNA and lncRNA) within the epigenetic modifications that may affect gene regulation of cell fate and differentiation, inflammation, and cancer [59,60,61,62,63,64,65,66]. Of note, miRNAs are small non-coding nucleotide sequences functioning by post-transcriptional regulation of gene expression. LncRNAs, on the other hand, are usually composed of >200 nucleobases, serving as scaffold for transcriptional factors [37]. The former is implicated in synapse formation, maturation, and dendritogenesis [67,68]. It has been reported that miR-9 and miR-9* are targets of the transcription factor RE1-silencing transcription factor (REST) [69]. This is an important factor eventually regulating neuronal development and differentiation by orchestrating TET3, the prominent neuronal TET isoform [70]. As for miRNA, lncRNAs are also in charge for modulating REST, as reported in a recent work describing the synergistic activity between REST and EZH2 in silencing target genes. EXH2 is in turn recruited by HOX transcript antisense RNA (HOTAIR), whose transcription is under control of an lncRNA [71].

As described above, epigenetics plays a key role in neuronal development and in neurodegenerative diseases. Herein, we will focus on the dysregulation of these epigenetic factors in Alzheimer’s disease (AD), Parkinson’s disease (PD), amyotrophic lateral sclerosis (ALS), and multiple sclerosis (MS), which share as common hallmarks neuroinflammation and a remarkable role of innate and adaptive immune cell populations in converging mechanisms of degeneration.

## 2. Epigenetic Alterations in Alzheimer’s Disease (AD)

AD has been classified as a primary amyloidosis. This family of diseases is characterized by accumulating fibrous proteins enriched with β-sheet secondary structures, generated by proteolysis of the amyloid precursor protein, encoded by a gene located on chromosome 21, and generally named β-amyloid or Aβ [72]. In AD, dense-core Aβ deposits have been detected on primary motors and visual cortices [73]. These account for the strong neuroinflammatory state depicted in AD, since Aβ plaques are recognized by microglia through pattern-recognition receptors (TLR1, TLR2, TLR4, and TLR6), CD14, CD47, α6β1 integrin, and scavenger receptors, eventually eliciting a strong glial activation and pro-inflammatory cytokines accumulation [74]. AD is also considered the most prevalent tauopathy, a term referring to neurodegenerative disorders associated with neuro- and glia-accumulating tau, a microtubule-associated phosphoprotein, promoting their stabilization and polymerization in axons [75]. Indeed, AD is classified as a secondary tauopathy, as the mutations characterizing AD mainly affect the presenilin genes (PSEN1 and PSEN2) and the amyloid precursor protein gene (APP), resulting in an altered amyloid metabolism [76]. Overall, macroscopic AD changes in frontal, temporal, and parietal lobes strictly correlate with the density of tau plaques, mainly composing the neurofibrillary tangles (NFTs) accumulated in neurons [77]. It is worth noticing a significant inflammatory response in AD is now established, which includes amoeboid reactive microglia and reactive astrogliosis surrounding the senile plaques [78]. Particularly, astrocytes are suggested as critical players in AD neuroinflammation and as master regulators of synapses formation, ion homeostasis, and neurovascular coupling [79,80,81]. Moreover, a substantial heterogeneity among astroglial cells in the CNS has been reported, suggesting region-specific profiles that further increase the complexity of the neuroinflammatory microenvironment [82].

Several studies have been carried out to investigate the role played by neuroinflammation in AD progression. In this regard, increased levels of IL-1β, IL-6, IL-8, IL-12, IL-18, TNF, and transforming growth factor-β (TGFβ) have been reported in AD patients compared to healthy subjects [2,83]. In general, two main concerns arise: (i) given the discrepancies of the studies regarding their inflammatory role, the value of these markers is questionable, and (ii) their concentration is also affected by systemic inflammation [2]. For this reason, recent research focused on the microglial activation markers chitinase-3-like protein 1 (YKL40), monocyte chemotactic protein 1 (MCP1), visinin-like protein-1 (VILIP1), and glial fibrillary acidic protein (GFAP) has found them to be upregulated in the cerebrospinal fluids (CSF) of AD patients [84]. Further strategies involve the use of positron emission tomography (PET) targeting translocator protein (TSPO), which is a marker of activated glial cells [85]. However, the development of a new generation of TSPO tracers has improved the signal-to-noise ratio but failed in distinguishing the contribution of different glial phenotypes responsible for neuroinflammation [2]. For this reason, new targets for PET tracers, including specific intracellular enzymes or molecules, are currently under investigation to achieve better information about different glial cell phenotypes involved in neuroinflammation [86,87]. It is worthy to mention that AD development is strictly correlated to changes in apolipoprotein E (APOE) expression, a lipid-binding protein regulating cholesterol transport in the brain. APOE is encoded as three different isoforms, namely ε2, ε3, and ε4 [88]. In the AD population, patients carrying one ε4 allele have a two–threefold increased AD risk, while those carrying two ε4 alleles have a 10–15-fold increased AD risk [88]. As already described, the expression of single isoforms may be related to epigenetic modifications. For this reason, epigenetic changes affecting APOE expression have been deeply investigated. While some reports did not describe any difference in APOE methylome in AD [89], other studies showed a decrease in APOE methylation [90,91]. Furthermore, other genes were investigated, including BDNF [92], glycogen synthase kinase 3 beta (GSK3β) [93], triggering receptor expressed on myeloid cells 2 (TREM2) [94], and ankyrin 1 (ANK1) [95]. However, these studies indicated that specific changes in gene methylation could be ruled out as the prominent factor driving AD pathology, overall prompting for the investigation of broader alteration. Corroborating this hypothesis, next-generation sequencing (NGS) analysis described a significant increase in CpG methylation within the hippocampus, temporal gyrus, entorhinal, dorsolateral prefrontal, and temporal cortex of AD patients [96,97,98]. Similarly, a significant decrease in methylation was reported in the prefrontal cortex and locus coeruleus [99]. Further analysis, performed by immunohistochemistry, revealed an enhanced DNA methylation in astrocytes, pyramidal neurons, and hippocampal CA1 neurons [100]. Interestingly, supporting the crucial role played by epigenetics in AD pathogenesis, Fetahu et al. identified 27 AD region-specific and 39 CpG site-specific epigenetic signatures which were independently validated across a clinical cohort. These modifications involve 5-methyl cytosine (5mC), 5-hydroxymethyl-cytosine (5hmC), and 5-formyl/carboxy-cytosine distribution in AD or AD-related genes or on genomic regions that are critical for proper neurodevelopment [101]. These findings might thus provide a first hint on the role of epigenetics modifications as a possible diagnostic marker for AD etiology.

AD pathogenesis is strictly related to a decline in mitochondrial fitness, resulting in an impaired reactive oxygen species (ROS) balance and increased apoptotic rate [102]. The epigenetic modifications affecting mitochondria, named “mitoepigentics”, turned out to be an interesting field of research, corroborated by results describing DNMT1 mitochondrial translocation, where DNMT3a, DNMT3b, TET1, and TET2 were already detected [103]. In this context, altered ROS levels have been linked to nuclear erythroid factor 2 (NRF2) activation, in turn inducing DNA demethylation [104].

Two independent studies reported that mitochondrial DNA (mtDNA), isolated from peripheral blood, showed an increase in CpG methylation in AD patients, eventually showing also DNMT3a A448G polymorphism [105,106].

**Figure 2:**
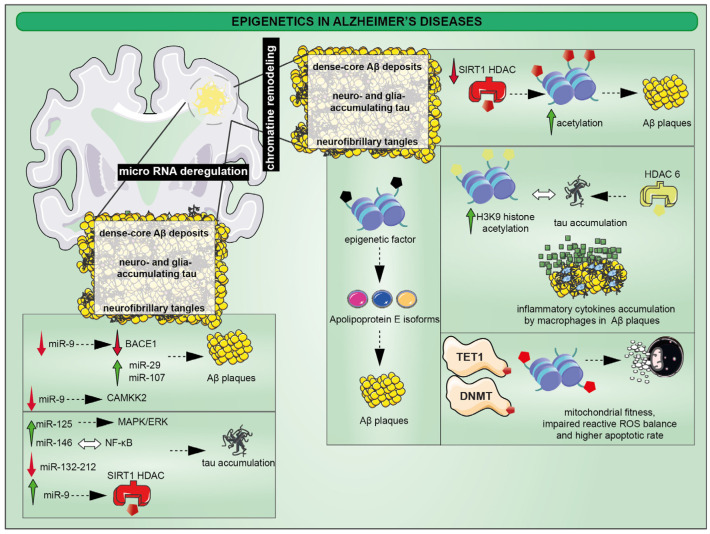
Accumulation of abnormal neurotic plaques and neurofibrillary tangles are the main pathological traits of AD. The first is related to the incorrect sequential cleavage of the amyloid precursor generating the fibrous protein enriched of β-sheet secondary structures. The second is the result of hyperphosphorylation of microtubule-associated tau protein in neurons leading to cytoskeletal changes. Among epigenetic changes, the acetylation processes lead to several effects. The reduction of Sirt1, a class III HDAC, determines a lack of histone deacetylation with downstream effects resulting in the development of Aβ plaques. However, inflammatory processes triggered by reactive microglia in plaques orchestrates tau hyperphosphorylation, where H3K9 acetylation by HDAC6 activity has been found to be implicated in the epigenic changes. Moreover, aberrant DNA methylation, especially DNMTs and TET, are associated to mitochondrial dysfunction causing the homeostatic loss of ROS balance which in turn is responsible for the apoptotic death of neurons. Finally, epigenetic changes also drive the formation of apolipoprotein E isoforms that show increased production and reduced degradation capacity of Aβ plaques; miR-9 is one the main miRNAs involved in AD pathogenesis, since its reduction determines a decrease of BACE1 and the activation of CAMKK2 which are responsible for Aβ plaques and p-tau, respectively. On the other hand, its upregulation control SIRT1 activity proves the correlation between miRNA and chromatin remodeling in AD pathological traits; MAPK/ERK activation is related to miR-125b for p-tau formation, whereas miR-146 expression is strictly linked to NF-κB. Other miRNAs, such as miR-132/-212 and the interaction between miR-29 and miR-107, directly correlate with Aβ plaques and p-tau accumulation.

As already discussed, epigenetics also includes a plethora of chemical modifications affecting nucleosome composition (Figure 2).

Interestingly, several reports have been published describing a loss of heterochromatin rate in the tau transgenic animal model, also mirrored in AD patients [107,108]. This outcome is strictly related to a decrease in histone acetylation rate, as described by two independent studies performing epigenome-wide association studies (EWAS) on postmortem AD brains [109,110]. As a result, 4162 different H3K27-acetylated peaks were identified comparing healthy and AD brain samples. Of note, these changes involve genes taking part in AD pathogenesis including APP, PSEN1, PSEN2, and MAPT, together with genes affecting neuronal activity such as GABA receptors activity and synaptic proteins [109]. H3K9 histone acetylation profiling, on the other hand, depicted an epigenome profile profoundly changed upon tau burdening. As a result, 5990 out of 26384 H3K9ac domains were changed in the dorsolateral prefrontal cortex of AD patients [110]. Besides these, some other epigenetic markers were also identified: lower levels of H4K16ac were identified in AD patients’ cortex [111], while an increase in H4K12ac has been linked to memory impairment [112]. Corroborating these data, further evidence supporting an increase in H3 and H4 acetylation in AD postmortem brains has been described [112]. As already discussed in the first section, chromatin acetylation level is orchestrated by HACs and HDAC activities. Interestingly, increased levels of class I HDAC2 and HDAC3 have been detected in brain regions related to the impairment in cognitive and synaptic functions [113]. In AD, enhanced levels of the class II HDAC6 have been described, in turn affecting the tubulin acetylation, eventually also orchestrating tau phosphorylation and the upcoming AD-linked inflammatory phenotype [114]. In agreement with these data, HDAC6 silencing rescues tau aggregation, thus helping neuronal survival [114]. Recent reports also focused on HDAC4, which may turn out to be an important player for memory formation and learning, as supported by data showing that HDAC4 overexpression enhances neuronal apoptotic phenomena [115]. Finally, class III HDACs, named sirtuins, have also been proven to be decreased in the AD parietal cortex, eventually accounting for Aβ accumulation [116,117].

It is worth noting that histone modifications also include the addition of methyl groups, operated by histone methyltransferases and histone demethylases, which have been reported to be dysregulated in the AD context [118]. An increase in H3K9 trimethylation, in synergy with euchromatic histone lysine methyltransferase 1 (EHMT1), has been found in postmortem AD brains. The outstanding role covered by histone methylation in AD pathogenesis has also been depicted in animal models, where histone methyltransferase G9a (HMT G9a) has been linked to mice cognitive features [119].

As mentioned above, a further layer of epigenetic modifications includes micro-RNA (miRNA). The goal of research plans, aiming at detecting miRNA regulation in AD pathogenesis, was mainly focused on their attractiveness as biomarkers, which may be detected either in the CNS or in the periphery [118]. To date, 61 different miRNAs targeting AD-related genes have been reported, eventually contributing to APP cleavage, Aβ metabolism, microtubule-associated protein tau (MAPT) regulation, and synaptic plasticity [120]. Regardless, data describing specific miRNAs are usually controversial, mostly due to the cohort heterogeneity. miR-9 downregulation, for instance, has been reported to be correlated to BACE1 downregulation, in turn promoting Aβ production and aggregation [121]. Furthermore, miR-9 is also in charge for calcium/calmodulin-dependent protein kinase 2 (CAMKK2) regulation [122]. As a result, the adenosine monophosphate-activated protein kinase (AMPK) pathway becomes active, enhancing the p-tau level. miR-9 upregulation, on the other hand, modulates transforming growth factor, β-induced (TGFBI), tripartite motif-containing 2 (TRIM2), and SIRT1 expression [123]. Similarly, there are several reports claiming the significative role covered by miR-146 in AD pathogenesis, which is unfortunately controversial [118]. It seems that miR-146 expression is strictly linked to nuclear factor kappa-B (NF-κB), in turn mitigating CNS inflammatory response by promoting miR-146 expression. In AD, a dysregulation of this loop leads to increased inflammation and degeneration. However, the amount of data produced so far described a general downregulation and upregulation of this miRNA in the serum, plasma, CSF, and CNS of AD subjects [124]. Despite the controversial evidence, several works have been aiming to investigate the role of different miRNAs in AD etiopathogenesis. Shioya and colleagues reported an interesting correlation standing in between miR-29 expression and BACE1 [125]. This gene is also regulated by the interaction with miR-107, as shown by Wang and colleagues [126]. Furthermore, miR-29 has been reported to target neuron navigator 3 (NAV3), whose role in AD pathogenesis is yet unknown, but it is upregulated in the frontal cortex of AD patients [125]. Further insights have linked miR-29 expression to B-cell lymphoma 2 (Bcl-2) Homology 3 (BH3)-only protein family activity. A decrease in miR-29, as reported in AD subjects, may eventually contribute to the increased apoptotic rate described in this neurodegenerative disease [127]. Bcl-2 also emerged as a possible miR-34a target. Enhanced expression of this miRNA could hamper Bcl-2 downstream, ultimately triggering neuronal apoptosis [128]. Further miRNAs in charge for orchestrating AD-related apoptotic events have been reported. miR-125b orchestrates the anti-apoptotic protein B-cell lymphoma-W (Bcl-W) expression, according to the work by Banzhaf-Strathmann and colleagues, eventually also affecting tau phosphorylation [129]. Corroborating this, elevated miR-125b expression correlates with tau hyperphosphorylation, following MAPK/ERK signaling activation [129]. Interestingly, in mice, miR-132/-212 deficiency also correlates with tau hyperphosphorylation [130]. MAPK cascade might also be affected by miR-181 expression, in turn mostly downregulated in AD CNS. Interestingly, a decrease in miR-181 expression, also targeting serine palmitoyl transferase long chain base subunit 1 (SPTLC1) positively correlates with Aβ accumulation [131]. Accumulation of Aβ and APP, typical of AD pathogenesis, has been reported to enhance the expression of inflammatory-associated miRNAs, including miR-155 in exosomes derived from AD subjects [132].

## 3. Epigenetic Alterations in Parkinson’s Disease (PD)

PD has been depicted as a neurodegenerative disorder affecting the dopaminergic nigrostriatal system, mainly disrupting dopaminergic neurons, eventually resulting in a defective dopamine transmission from the substantia nigra pars compacta to the caudate putamen. Therefore, the main symptoms characterizing this disease include tremor at rest, rigidity, bradykinesia, and postural instability. In addition, PD pathogenesis involves extra-nigral dopaminergic, serotoninergic, and cholinergic neurons, eventually resulting in further symptoms such as anosmia, sleep disorders, dementia, and depression [133]. The etiopathology of the disease is strictly linked to mutations affecting PARK in 5- to 10% of PD cases, the gene encoding for alpha-synuclein (α-Syn). Additional risk factors accounting for PD onset include toxins, pesticides, heavy metals, traumatic lesions, and bacterial or viral infections [134]. Interestingly, the inflammatory component within this context turns out to play a crucial role, since there have been reports claiming that neurotrophic pathogens may reach the basal ganglia, exploiting the nasal and intestinal mucosa, eventually triggering a consistent inflammatory response driving PD pathogenesis [135]. Moreover, PD progression involves damage-associated molecular pattern (DAMP) released by neurons, proinflammatory mediators secreted by astrocytes, misfolded or aggregated proteins including α-Syn trigger microglia polarization towards a proinflammatory phenotype [136]. Of note, DAMP release may be responsible for the NADPH oxidase (NOX)-enhanced expression along with an increase in ROS and nitric oxide species generation. Together with this, reactive microglia are characterized by a proinflammatory secretome depicted by release of cytokines, i.e., IL-1β, IL-6, TNF, with chemokines and bioactive lipids eventually reinforcing the already inflamed microenvironment. As a result, such a cellular state might impair the blood–brain barrier (BBB), eventually resulting in an increased leukocyte infiltration further boosting the local inflammatory response [137]. Supporting this scenario, two independent studies showed an increase in cellular death rate of cultured dopaminergic neurons upon exposure to a conditioned media derived either from M1-like microglia and lipopolysaccharide (LPS)-treated glial cells [138,139]. Furthermore, several in vivo studies have been reported in agreement with the previously described models: strial injection of the catecholaminergic neurotoxin 6-hydroxydopamine (6-OHDA) triggers microgliosis, characterized by an increase of IL-1β, IL-6, TNF, and IFNγ and eventually leading to astrogliosis and dopaminergic cell death [140]. Similarly, 1-methyl-4-phenyl-1,2,3,6-tetrahydropyridine (MPTP) is used to recapitulate PD pathogenesis triggering dopaminergic neuronal death in primates and mice by ATP depletion and enhancing ROS levels [141]. Despite the induction of the neuronal death, both 6-OHDA and MPTP fail in mimicking the main feature of PD: the accumulation of α-Syn in Lewy bodies. For this reason, recent advances in preclinical models of PD have used direct nigrostriatal inoculation of preformed fibrils of α-Syn or by delivery of α-Syn-expressing adeno-associated viral vectors [142,143]. As previously mentioned, the inflammatory microenvironment characterizing PD also involves different cellular species including astrocytes, which express high levels of the PD-related genes PARK7, SNCA, PLA2G6, ATP13A2, LRRK2, GBA, PINK1, and PARK2 [144]. Furthermore, α-Syn inclusions have been reported to also accumulate in astrocytes, probably as a consequence of neuronal transmission [145]. Here, α-Syn accumulates within lysosomes, where it might drive autophagic degradation. Moreover, further processes involve the acquisition of a senescent-associated secretory phenotype by astrocytes along PD progression, eventually enhancing the microenvironmental inflammatory profile, as supported by data showing that conditioned media, isolated by senescent astrocytes, trigger dopaminergic neuronal death in an in vitro model [146]. The mechanistic process behind this effect may be linked to TNF, in turn released by activated astrocytes and binding specific receptors on dopaminergic neurons such as TNFR1 and 2, ultimately triggering apoptosis [147].

**Figure 3:**
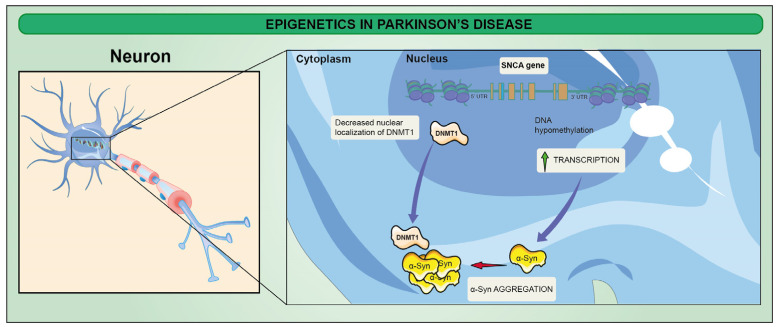
Epigenetics in PD. DNA hypomethylation of SNCA is associated with increase of SNCA expression. DNMT1 (a regulator of DNA methylation) plays an important role in SNCA expression. DNMT1 is mainly located in the nucleus of neurons, and α-Syn aggregation leads to cytoplasmic sequestration of DNMT1, resulting in decreased nuclear localization of DNMT1, which is likely to be involved in the pathogenesis of PD.

From an epigenetic point of view, most of the efforts focused on evaluating the methylome profile of the α-Syn-encoding gene (SNCA) have been so far contradictory. However, important data emerged by application of DNA methyl transferase (DNMT) inhibitors, eventually repressing SNCA expression, which is hypomethylated during neuronal differentiation towards the dopaminergic phenotype [148]. Globally, a 30% reduction in DNA methylation has been observed as a consequence of the reduction in nuclear DNMT1, but, to date, further inquiries investigating the link between changes in DNA methylome and PD progression are needed (Figure 3) [149].

Histone acetylation, on the other hand, has been widely discussed over the past years. Indeed, an increase in HAT activity over HDAC has been reported, eventually drawing a scenario in which PD-associated genes might turn out to be hyperacetylated. This is supported by two studies reporting a decrease of p300 acetylating mediated by α-Syn. In particular, in vitro cellular studies reported an increase in histone acetylation following cytotoxic stress mediated by LPS, α-Syn, and MPTP. Furthermore, it has been reported that single nucleotide polymorphism (SNP) on intron 4 of SNCA leads to a lack of EMX2/NKX6.1 recruitment, in turn mediating SNCA repression and triggering a hyperacetylation of H3K27, otherwise methylated as a consequence of HDAC recruitment [150,151]. This hypothesis was further corroborated by Mittal et al., eventually showing a repression of SNCA as a consequence of β2-Adrenoreceptor (β2AR) administration in vitro, in turn promoting HDAC association to SNCA [152]. Further evidence has been reported recently by Toker and colleagues. Their work described a significant increase in H3K27 acetylation in the prefrontal cortex of individuals with PD. This marker of active transcription was also strongly associated to PD hallmarks including SNCA, MAPT, APP, PRKN, PARK7, FBOX7, and POLG [153]. The opposite mechanism, H3K27 methylation, is mediated also by Ezh 1/2. Interestingly, the loss of these proteins leads to degeneration of dopaminergic neurons [151]. Further evidence has been reported about Sirt1, a class III HDAC, in charge of deacetylation of the NfKB gene, thus proposing the epigenetics drift as a possible player promoting the proinflammatory microenvironment [154]. To date, further studies are still required in order to investigate this interconnection, which could lead to new therapeutic approaches towards PD neurodegeneration.

## 4. Epigenetics in Amyotrophic Lateral Sclerosis (ALS)

ALS has been characterized as a fatal neurodegenerative disease leading to a progressive loss of the upper and lower motoneurons eventually occurring in middle to late age and resulting in death within three to five years [155]. This neuropathology can be distinguished based on the involvement of upper or lower motoneurons, while further classifications involve ALS neuroanatomical distribution (i.e., spinal and bulbar ALS) and sporadic (s)ALS versus familiar (f)ALS [1]. ALS patients display atrophy of the anterior spinal nerve roots, motor cortex, and spinal cord, while ALS patients showing clinical signs of dementia also suffer from focal atrophy of the temporal and frontal lobes [156]. Interestingly, these morbidity factors are also characterized by TDP-43 inclusions, eventually evaluated by immunohistochemistry assay [157]. Current treatments have limited effects, and despite intensive effort, the pathogenic mechanisms underlying this disease are still poorly understood [158,159], although new imaging and neuropathological data have indicated the involvement of the non-motor neuroaxis in the pathophysiology of the disease. Interestingly, it has been reported that damaged motoneurons in ALS may release several misfolded proteins, including SOD1, in turn eliciting microglia-mediated inflammatory response, as showed by PET analysis on SOD1-mutated mice models [160]. Recent reports also describe ATP release upon neuronal death as one of the factors activating microglia through the activation of the ionotropic P2X and metabotropic P2Y purinergic receptors [161]. In line with this evidence, increased P2X7 levels have been detected in postmortem ALS spinal cord, and upregulation of P2X4, P2X7, and P2Y7 has been reported in SOD1-mutated microglia. Consequently, ATP hydrolysis impairment triggers TNF and cyclooxygenase-2 (COX-2) production [161]. Apolloni and colleagues, on the other hand, depicted a more complex scenario involving P2X7 [162]. Their work reported a worsening ALS progression following constitutive P2X7 depletion in end-stage (23-week) SOD1-mutated mice. As a result, they recorded an increased motoneuronal loss in parallel with an increase in nicotinamide adenine dinucleotide phosphate oxidase 2 (NOX2) and inducible nitric oxide synthase in the lumbar spinal cord of SOD1 mutated mice [162]. In agreement with these data, the usage of P2X7 antagonist Brilliant Blue G enhanced motoneuron survival [163]. Overall, it seems that P2X7 plays a dual action depending on a time window along ALS progression. Like microglia, astrocytes are also involved in ALS pathogenesis. However, it is still unclear how ALS-related misfolded or mutated proteins are linked to astrocytes dysfunction [164]. In this respect, it has been described that in sALS and fALS, astrocytes are no longer efficient in clearing excess glutamate [164]. Moreover, several astrocyte metabolic dysfunctions have been observed, including mitochondrial defects and lactate efflux dysfunctions. The deficiency in mitochondrial respiration may be linked to epigenetic changes affecting mitochondrial DNA (mtDNA) [165]. Increasing evidence suggests that changes in DNA methylation and hydroxylation affect both mtDNA replication and gene expression levels. It was noted that the mtDNA copy number was increased in SOD1- or C9orf72-mutated ALS patients [166]. Consequently, these defects exacerbate motoneuronal death through a caspase-independent form of programmed cell death called necroptosis. For this reason, targeting the main proteins orchestrating necroptosis may represent a promising strategy towards development of efficient ALS drugs [167]. The pathological processes that lead to neuronal death in ALS are not yet completely understood, but oxidative stress, impaired axonal transport, protein and RNA aggregation, excitotoxicity, neuroinflammation, and mitochondrial dysfunction have been reported as critical degenerative processes characterizing its neuropathology. In fact, recent evidence suggests that epigenetic modifications to the mitochondrial genome could also contribute to neurodegeneration [165].

**Figure 4:**
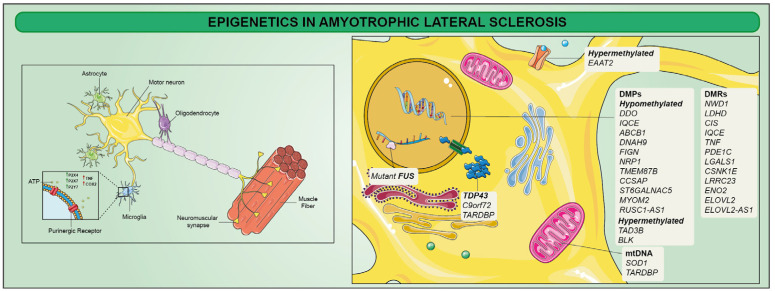
Mutations in several genes that have been implicated in the pathophysiology of amyotrophic lateral sclerosis (ALS) have been reported as triggers for disease neuroinflammation and neurodegeneration. TDP-43 inclusions, SOD1, C9orf72, TARDBP, and FUS gene mutations have been identified as further causative or highly associated with ALS pathogenesis. Moreover, differentially methylated positions (DMPs) and differentially methylated regions (DMRs) of specific genes have been suggested as ALS contributors. Other mechanisms are largely correlated with ALS: for instance, microglia-mediated inflammatory response can directly contribute to motor neuron dysfunction and death, producing inflammatory mediators and reactive oxygen and nitrogen species.

A subset of ALS patients harbors mutations in their genes that play different roles in neuronal functioning [168,169]. Within this context, the first gene associated with ALS was SOD1, identified in 1993. Furthermore, by early 2014, more than 20 genes including C9orf72, TARDBP, and FUS have been identified as further causative or highly associated with ALS pathogenesis. However, mutations in these genes account for approximately two-thirds of all fALS patients and approximately 10% of sALS, where genetic factors can only explain a small proportion of patients. In addition to genetic factors, several studies suggested that environmental factors may account for ALS progression [170,171,172]. As a result, these factors may drive epigenetic modifications, eventually explaining disease onset and progression. Several authors reported the identification of alterations in global 5hmC rate associated with ALS [173]. Moreover, the 5mC rate was also affected in spinal cord genes exhibiting concordant mRNA expression overrepresented in functional categories implicated in ALS. Moreover, 34 significant differentially methylated positions (DMPs) in whole blood from sALS patients were recently identified [174]. These included 13 genes: while TAD3B and BLK were hypermethylated, DDO, IQCE, ABCB1, DNAH9, FIGN, NRP1, TMEM87B, CCSAP, ST6GALNAC5, MYOM2, and RUSC1-AS1 were hypomethylated. These authors also identified 12 differentially methylated regions (DMRs) related to 12 genes (NWD1, LDHD, CIS, IQCE, TNF, PDE1C, LGALS1, CSNK1E, LRRC23, ENO2, ELOVL2, and ELOVL2-AS1) (Figure 4) [174].

Furthermore, ALS patients also displayed decreased SOD1, FUS, and VEGF methylation on promoter regions [175], synergistically with hypermethylation of promoters of human glutamate transporter EAAT2, which has been suggested as an ALS contributor [176]. SOD1-mutated patients also showed a decrease in D-loop methylation levels suggesting a compensatory mechanism for mtDNA, eventually showing a hypermethylated profile [177]. Taken together, these epigenetic changes identify key disease pathways that are therapeutically testable, which could potentially lead to the development of better treatments for ALS patients.

## 5. Epigenetics in Multiple Sclerosis (MS)

MS is an inflammatory autoimmune disorder of the CNS with a significant increased incidence in women, with a female to male ratio of 3:1 [178]. A number of factors accounting for MS onset must be considered. For instance, increasing prevalence of MS with equator distance and the incidence of it being related to a given geographic area [179]. Evidence revealed that immigrants who move later in life conserved the risk of the original geographic area, and the change in risk level may not appear until the next generation. Importantly, those who move in early childhood tend to acquire the new geographic risk themselves [179]. MS also has a familiar recurrence rate, with 3% risk in first-degree relatives (siblings, 5%; parents, 2%; and children, 2%) and 1% risk in second-degree and third-degree relatives, suggesting genetic factors in determining familiar clustering and susceptibility [180]. Similarly, active smoking seems to play a role with a 1.8 ratio for men and 1.4 ratio for women with MS versus healthy individuals [181].

The major early driver of tissue damage in MS lesions is the migration of autoreactive lymphocytes from the periphery, which cross the BBB to invade the CNS. Accumulation of T and B lymphocytes, plasma cells, and activated mononuclear phagocytes triggers the secretion of pro-inflammatory cytokines, amplifying the immune response through the recruitment of naïve microglia [179]. Inflammatory mediators such as cytokines (i.e., IL-12, IFN-γ, and TNF), nitric oxide, and free radicals, produced by infiltrating cells and resident microglia, play a critical role in demyelination, contributing to oligodendrocyte loss and degenerative axonal pathology. Demyelination observed in MS patients is typically the completion of a direct insult to the oligodendrocytes (OLs) [182,183]. In physiological conditions, OLs wrap segments of axons and produce myelin. Neurons, taking advantages from this intimate contact with OLs, can rapidly conduct stimuli via saltatory conduction of action potentials that propagate throughout the so-called nodes of Ranvier. In demyelinating conditions, the disruption of these structures impairs the conductivity but also affects axonal trophism, leading to neurodegeneration as a chronic condition [179]. While axonal loss and neurodegeneration coexist with demyelination in the progressive stage of the disease, compensatory mechanisms are initiated to overcome the chronic damage. Among these, oligodendrocyte precursor cells (OPCs) recruiting is important as these cells can migrate into a demyelinated area and differentiate into mature myelinating OLs [184]. However, these mechanisms seem largely inadequate, and remyelination is less successful after cycles of demyelination and remyelination, probably due to the exhaustion of tissue repair capacity. Such a phenomenon led to the hypothesis of potential age-dependent mechanisms hampering successful regenerative mechanisms in progressive (P)-MS. Indeed, the ability to recover from the relapse decreases with age, with a concomitant increased risk to develop the progressive form of the disease [185,186,187,188].

Despite the therapeutic effects of disease-modifying agents in relapsing–remitting (RR)-MS patients, P-MS still lacks effective treatments. Such a stark contrast is partially dependent on the difficulties in clarifying the complex neuroinflammatory processes of MS, which couple demyelination and neurodegeneration. Interestingly, important hints have been reported about vitamin D as a protective factor in large epidemiologic studies. Healthy controls exhibit higher serum levels of 25(OH)D3 and 1,25(OH)2D3 (the active form of vitamin D) than RR-MS patients. Moreover, these patients present lower serum levels of vitamin D during relapses compared to the levels during remissions. The molecular mechanisms underlying this evidence still need to be fully elucidated, but it has been reported that 1,25(OH)2D3 supports the induction of CD4^+^ factor forkhead box P3 (FOXP3^+^) regulatory T cells by rendering of tolerogenic dendritic cells [189]. Taken together, this body of evidence suggests an interplay between environmental and genetic factors in the pathogenesis of MS.

**Figure 5:**
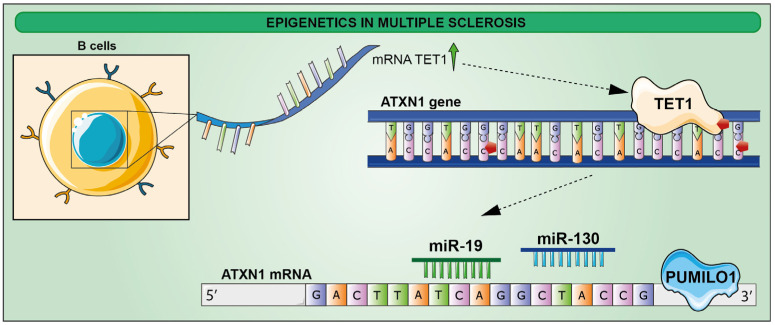
Schematic representation of epigenetic modifications in B cells at the clinical onset of MS. ATXN1 gene is hypomethylated in four sites by TET1, of which mRNA is upregulated. PUM1, with miR-19 and miR-130, stabilizes ATXN1 mRNA, leading to the increase in ATXN1 protein levels.

Although the etiology of the disease is unknown, an epigenetic component appears to influence the onset and progression of the disease, and in addition to DNA methylation and miRNA-based gene expression regulation, histone modification has also been implicated in MS pathogenesis. Four hypomethylated sites in the ATXN1 genomic sequence of B cells at the clinical onset of the disease have been recently described. These changes might be mediated by TET1, whose mRNA has been found to be upregulated following RNA-seq analysis [190]. These data were further supported by studies on preclinical models showing an increase in ataxin-1 level, resulting from an enhanced ATXN1 mRNA [191]. Mechanistic insights have been gained showing that the RNA-binding protein PUMILO1 (PUM1) orchestrates ataxin-1 levels by increasing Atxn1 mRNA stability through its interaction with mRNA 3′UTR, along with miR-19 and miR-130, in turn proposed to mediate Atxn1 mRNA stability as well (Figure 5) [192,193].

Since DNA methylation has been widely reported to be linked to cell division and is likely to replicative senescence potential [194,195], it has been investigated whether accelerated aging in MS glia could be attributed to increased proliferation. Recent publications indicate a form of exhausted glial cells which are sustained by repeated damage to oligodendrocytes and myelin [196,197] and by the elevated demand for debris uptake by microglia [198], tissue repair by astrocytes, and remyelination by oligodendrocytes [199]. In the MS context, it has been recently described that glial cells of normal-appearing white matter (NAWM) in MS patients undergo genome-wide DNA methylation changes, correlating with transcriptional differences in genes involved in cytoskeleton organization, cell signaling, molecule transports, neuroinflammation, cell motility, and metabolic processes compared to controls [200]. This is possibly a consequence of KDM1A (LSD1) action, a flavin adenine dinucleotide-dependent amine oxidase located in the nucleus, acting as a histone-modifying enzyme [1], eventually recruited to repressive transcription complexes.

Taken together, these works force future research lines to consider the epigenetic component in the MS scenario, which could play a pivotal role to develop new therapeutic approaches.

## 6. Perspectives

This work was intended to review the current literature about the epigenetic changes so far reported about neuroinflammatory disorders. Unfortunately, few works today have been carried out to identify a potential epigenetic marker that could represent either a new target for the development of novel drugs or to improve the current diagnostic strategies. For this reason, further studies are needed in order to discriminate further epigenetic players that could become discriminating in these neuropathologies.

## References

[1] Dugger B.N., Dickson D.W. (2017). Pathology of Neurodegenerative Diseases. Cold Spring Harb. Perspect. Biol..

[2] Leng F., Edison P. (2021). Neuroinflammation and microglial activation in Alzheimer disease: Where do we go from here?. Nat. Rev. Neurol..

[3] Ghezzi P., Bernardini R., Giuffrida R., Bellomo M., Manzoni C., Comoletti D., Di Santo E., Benigni F., Mennini T. (1998). Tumor necrosis factor is increased in the spinal cord of an animal model of motor neuron degeneration. Eur. Cytokine Netw..

[4] Vicario N., Castrogiovanni P., Imbesi R., Giallongo S., Mannino G., Furno D.L., Giuffrida R., Zappala A., Li Volti G., Tibullo D. (2022). GJA1/CX43 High Expression Levels in the Cervical Spinal Cord of ALS Patients Correlate to Microglia-Mediated Neuroinflammatory Profile. Biomedicines.

[5] Vicario N., Parenti R. (2022). Connexins Signatures of the Neurovascular Unit and Their Physio-Pathological Functions. Int. J. Mol. Sci..

[6] Mannino G., Russo C., Maugeri G., Musumeci G., Vicario N., Tibullo D., Giuffrida R., Parenti R., Lo Furno D. (2022). Adult stem cell niches for tissue homeostasis. J. Cell Physiol..

[7] Mannino G., Vicario N., Parenti R., Giuffrida R., Lo Furno D. (2020). Connexin expression decreases during adipogenic differentiation of human adipose-derived mesenchymal stem cells. Mol. Biol. Rep..

[8] Spitale F.M., Vicario N., Rosa M.D., Tibullo D., Vecchio M., Gulino R., Parenti R. (2020). Increased expression of connexin 43 in a mouse model of spinal motoneuronal loss. Aging.

[9] Kasongo D.W., de Leo G., Vicario N., Leanza G., Legname G. (2020). Chronic alpha-Synuclein Accumulation in Rat Hippocampus Induces Lewy Bodies Formation and Specific Cognitive Impairments. eNeuro.

[10] Gulino R., Vicario N., Giunta M.A.S., Spoto G., Calabrese G., Vecchio M., Gulisano M., Leanza G., Parenti R. (2019). Neuromuscular Plasticity in a Mouse Neurotoxic Model of Spinal Motoneuronal Loss. Int. J. Mol. Sci..

[11] Vicario N., Calabrese G., Zappala A., Parenti C., Forte S., Graziano A.C.E., Vanella L., Pellitteri R., Cardile V., Parenti R. (2017). Inhibition of Cx43 mediates protective effects on hypoxic/reoxygenated human neuroblastoma cells. J. Cell Mol. Med..

[12] Vicario N., Turnaturi R., Spitale F.M., Torrisi F., Zappala A., Gulino R., Pasquinucci L., Chiechio S., Parenti C., Parenti R. (2020). Intercellular communication and ion channels in neuropathic pain chronicization. Inflamm. Res..

[13] Lo Furno D., Mannino G., Giuffrida R., Gili E., Vancheri C., Tarico M.S., Perrotta R.E., Pellitteri R. (2018). Neural differentiation of human adipose-derived mesenchymal stem cells induced by glial cell conditioned media. J. Cell Physiol..

[14] Mannino G., Gennuso F., Giurdanella G., Conti F., Drago F., Salomone S., Furno D.L., Bucolo C., Giuffrida R. (2020). Pericyte-like differentiation of human adipose-derived mesenchymal stem cells: An in vitro study. World J. Stem Cells.

[15] Calabrese G., Giuffrida R., Lo Furno D., Parrinello N.L., Forte S., Gulino R., Colarossi C., Schinocca L.R., Giuffrida R., Cardile V. (2015). Potential Effect of CD271 on Human Mesenchymal Stromal Cell Proliferation and Differentiation. Int. J. Mol. Sci..

[16] Panto M.R., Cicirata F., Angaut P., Parenti R., Serapide F. (1995). The projection from the primary motor and somatic sensory cortex to the basilar pontine nuclei. A detailed electrophysiological and anatomical study in the rat. J. Hirnforsch..

[17] Fagone E., Conte E., Gili E., Fruciano M., Pistorio M.P., Lo Furno D., Giuffrida R., Crimi N., Vancheri C. (2011). Resveratrol inhibits transforming growth factor-beta-induced proliferation and differentiation of ex vivo human lung fibroblasts into myofibroblasts through ERK/Akt inhibition and PTEN restoration. Exp. Lung Res..

[18] Gijselinck I., Van Mossevelde S., van der Zee J., Sieben A., Engelborghs S., De Bleecker J., Ivanoiu A., Deryck O., Edbauer D., Zhang M. (2016). The C9orf72 repeat size correlates with onset age of disease, DNA methylation and transcriptional downregulation of the promoter. Mol. Psychiatry.

[19] Hinz F.I., Geschwind D.H. (2017). Molecular Genetics of Neurodegenerative Dementias. Cold Spring Harb. Perspect. Biol..

[20] Tcw J., Goate A.M. (2017). Genetics of beta-Amyloid Precursor Protein in Alzheimer’s Disease. Cold Spring Harb. Perspect. Med..

[21] Ghasemi M., Brown R.H. (2018). Genetics of Amyotrophic Lateral Sclerosis. Cold Spring Harb. Perspect. Med..

[22] Voglein J., Kostova I., Arzberger T., Roeber S., Schmitz P., Simons M., Ruf V., Windl O., Herms J., Dieterich M. (2021). First symptom guides diagnosis and prognosis in neurodegenerative diseases-a retrospective study of autopsy proven cases. Eur. J. Neurol..

[23] Berger S.L., Kouzarides T., Shiekhattar R., Shilatifard A. (2009). An operational definition of epigenetics. Genes Dev..

[24] Giallongo S., Lo Re O., Vinciguerra M. (2019). Macro Histone Variants: Emerging Rheostats of Gastrointestinal Cancers. Cancers.

[25] Giallongo S., Lo Re O., Lochmanova G., Parca L., Petrizzelli F., Zdrahal Z., Mazza T., Vinciguerra M. (2021). Phosphorylation within Intrinsic Disordered Region Discriminates Histone Variant macroH2A1 Splicing Isoforms-macroH2A1.1 and macroH2A1.2. Biology.

[26] Giallongo S., Rehakova D., Biagini T., Lo Re O., Raina P., Lochmanova G., Zdrahal Z., Resnick I., Pata P., Pata I. (2022). Histone Variant macroH2A1.1 Enhances Nonhomologous End Joining-dependent DNA Double-strand-break Repair and Reprogramming Efficiency of Human iPSCs. Stem Cells.

[27] Giallongo S., Rehakova D., Raffaele M., Lo Re O., Koutna I., Vinciguerra M. (2021). Redox and Epigenetics in Human Pluripotent Stem Cells Differentiation. Antioxid. Redox Signal..

[28] Hu B., Won H., Mah W., Park R.B., Kassim B., Spiess K., Kozlenkov A., Crowley C.A., Pochareddy S., Psych E.C. (2021). Neuronal and glial 3D chromatin architecture informs the cellular etiology of brain disorders. Nat. Commun..

[29] Mannino G., Cristaldi M., Giurdanella G., Perrotta R.E., Lo Furno D., Giuffrida R., Rusciano D. (2021). ARPE-19 conditioned medium promotes neural differentiation of adipose-derived mesenchymal stem cells. World J. Stem Cells.

[30] Lo Furno D., Mannino G., Pellitteri R., Zappala A., Parenti R., Gili E., Vancheri C., Giuffrida R. (2018). Conditioned Media From Glial Cells Promote a Neural-Like Connexin Expression in Human Adipose-Derived Mesenchymal Stem Cells. Front. Physiol..

[31] Lo Furno D., Pellitteri R., Graziano A.C., Giuffrida R., Vancheri C., Gili E., Cardile V. (2013). Differentiation of human adipose stem cells into neural phenotype by neuroblastoma- or olfactory ensheathing cells-conditioned medium. J. Cell Physiol..

[32] Lo Furno D., Mannino G., Giuffrida R. (2018). Functional role of mesenchymal stem cells in the treatment of chronic neurodegenerative diseases. J. Cell Physiol..

[33] Russo C., Mannino G., Patane M., Parrinello N.L., Pellitteri R., Stanzani S., Giuffrida R., Lo Furno D., Russo A. (2021). Ghrelin peptide improves glial conditioned medium effects on neuronal differentiation of human adipose mesenchymal stem cells. Histochem. Cell Biol..

[34] Lo Furno D., Mannino G., Cardile V., Parenti R., Giuffrida R. (2016). Potential Therapeutic Applications of Adipose-Derived Mesenchymal Stem Cells. Stem Cells Dev..

[35] Mannino G., Russo C., Longo A., Anfuso C.D., Lupo G., Lo Furno D., Giuffrida R., Giurdanella G. (2021). Potential therapeutic applications of mesenchymal stem cells for the treatment of eye diseases. World J. Stem Cells.

[36] Lo Furno D., Graziano A.C., Caggia S., Perrotta R.E., Tarico M.S., Giuffrida R., Cardile V. (2013). Decrease of apoptosis markers during adipogenic differentiation of mesenchymal stem cells from human adipose tissue. Apoptosis.

[37] Hwang J.Y., Aromolaran K.A., Zukin R.S. (2017). The emerging field of epigenetics in neurodegeneration and neuroprotection. Nat. Rev. Neurosci..

[38] Miller C.A., Sweatt J.D. (2007). Covalent modification of DNA regulates memory formation. Neuron.

[39] Day J.J., Sweatt J.D. (2010). DNA methylation and memory formation. Nat. Neurosci..

[40] Feng J., Zhou Y., Campbell S.L., Le T., Li E., Sweatt J.D., Silva A.J., Fan G. (2010). Dnmt1 and Dnmt3a maintain DNA methylation and regulate synaptic function in adult forebrain neurons. Nat. Neurosci..

[41] Hayaishi O. (1969). Enzymic hydroxylation. Annu. Rev. Biochem..

[42] Ploumakis A., Coleman M.L. (2015). OH, the Places You’ll Go! Hydroxylation, Gene Expression, and Cancer. Mol. Cell.

[43] Tahiliani M., Koh K.P., Shen Y., Pastor W.A., Bandukwala H., Brudno Y., Agarwal S., Iyer L.M., Liu D.R., Aravind L. (2009). Conversion of 5-methylcytosine to 5-hydroxymethylcytosine in mammalian DNA by MLL partner TET1. Science.

[44] Kaas G.A., Zhong C., Eason D.E., Ross D.L., Vachhani R.V., Ming G.L., King J.R., Song H., Sweatt J.D. (2013). TET1 controls CNS 5-methylcytosine hydroxylation, active DNA demethylation, gene transcription, and memory formation. Neuron.

[45] Horvath S. (2013). DNA methylation age of human tissues and cell types. Genome Biol..

[46] Hannum G., Guinney J., Zhao L., Zhang L., Hughes G., Sadda S., Klotzle B., Bibikova M., Fan J.B., Gao Y. (2013). Genome-wide methylation profiles reveal quantitative views of human aging rates. Mol. Cell.

[47] Levine M.E., Lu A.T., Quach A., Chen B.H., Assimes T.L., Bandinelli S., Hou L., Baccarelli A.A., Stewart J.D., Li Y. (2018). An epigenetic biomarker of aging for lifespan and healthspan. Aging.

[48] Marioni R.E., Shah S., McRae A.F., Chen B.H., Colicino E., Harris S.E., Gibson J., Henders A.K., Redmond P., Cox S.R. (2015). DNA methylation age of blood predicts all-cause mortality in later life. Genome Biol..

[49] Grodstein F., Lemos B., Yu L., Iatrou A., De Jager P.L., Bennett D.A. (2020). Characteristics of Epigenetic Clocks Across Blood and Brain Tissue in Older Women and Men. Front. Neurosci..

[50] Shireby G.L., Davies J.P., Francis P.T., Burrage J., Walker E.M., Neilson G.W.A., Dahir A., Thomas A.J., Love S., Smith R.G. (2020). Recalibrating the epigenetic clock: Implications for assessing biological age in the human cortex. Brain.

[51] Raneros A.B., Bernet C.R., Florez A.B., Suarez-Alvarez B. (2021). An Epigenetic Insight into NLRP3 Inflammasome Activation in Inflammation-Related Processes. Biomedicines.

[52] Fang P., Chen C., Zheng F., Jia J., Chen T., Zhu J., Chang J., Zhang Z. (2021). NLRP3 inflammasome inhibition by histone acetylation ameliorates sevoflurane-induced cognitive impairment in aged mice by activating the autophagy pathway. Brain Res. Bull..

[53] Heneka M.T., Kummer M.P., Stutz A., Delekate A., Schwartz S., Vieira-Saecker A., Griep A., Axt D., Remus A., Tzeng T.C. (2013). NLRP3 is activated in Alzheimer’s disease and contributes to pathology in APP/PS1 mice. Nature.

[54] Wang L., Chen K., Wan X., Wang F., Guo Z., Mo Z. (2017). NLRP3 inflammasome activation in mesenchymal stem cells inhibits osteogenic differentiation and enhances adipogenic differentiation. Biochem. Biophys. Res. Commun..

[55] Baardman J., Licht I., de Winther M.P., Van den Bossche J. (2015). Metabolic-epigenetic crosstalk in macrophage activation. Epigenomics.

[56] Fischer A., Sananbenesi F., Wang X., Dobbin M., Tsai L.H. (2007). Recovery of learning and memory is associated with chromatin remodelling. Nature.

[57] Levenson J.M., O’Riordan K.J., Brown K.D., Trinh M.A., Molfese D.L., Sweatt J.D. (2004). Regulation of histone acetylation during memory formation in the hippocampus. J. Biol. Chem..

[58] Bourtchouladze R., Lidge R., Catapano R., Stanley J., Gossweiler S., Romashko D., Scott R., Tully T. (2003). A mouse model of Rubinstein-Taybi syndrome: Defective long-term memory is ameliorated by inhibitors of phosphodiesterase 4. Proc. Natl. Acad. Sci. USA.

[59] Gulino R., Forte S., Parenti R., Memeo L., Gulisano M. (2015). MicroRNA and pediatric tumors: Future perspectives. Acta Histochem..

[60] Stevanovic M., Stanisavljevic Ninkovic D., Mojsin M., Drakulic D., Schwirtlich M. (2022). Interplay of SOX transcription factors and microRNAs in the brain under physiological and pathological conditions. Neural Regen. Res..

[61] Zhu Y., Zhang X., Yang K., Shao Y., Gu R., Liu X., Liu H., Liu Y., Zhou Y. (2022). Macrophage-derived apoptotic vesicles regulate fate commitment of mesenchymal stem cells via miR155. Stem Cell Res. Ther..

[62] Pounders J., Hill E.J., Hooper D., Zhang X., Biesiada J., Kuhnell D., Greenland H.L., Esfandiari L., Timmerman E., Foster F. (2022). MicroRNA expression within neuronal-derived small extracellular vesicles in frontotemporal degeneration. Medicine.

[63] Sufianov A., Begliarzade S., Ilyasova T., Xu X., Beylerli O. (2022). MicroRNAs as potential diagnostic markers of glial brain tumors. Noncoding RNA Res..

[64] Li W., Shan B.Q., Zhao H.Y., He H., Tian M.L., Cheng X., Qin J.B., Jin G.H. (2022). MiR-130a-3p regulates neural stem cell differentiation in vitro by targeting Acsl4. J. Cell Mol. Med..

[65] Estrada-Meza C., Torres-Copado A., Loreti Gonzalez-Melgoza L., Ruiz-Manriquez L.M., De Donato M., Sharma A., Pathak S., Banerjee A., Paul S. (2022). Recent insights into the microRNA and long non-coding RNA-mediated regulation of stem cell populations. 3 Biotech.

[66] Naseer S., Abelleira-Hervas L., Savani D., de Burgh R., Aleksynas R., Donat C.K., Syed N., Sastre M. (2022). Traumatic Brain Injury Leads to Alterations in Contusional Cortical miRNAs Involved in Dementia. Biomolecules.

[67] Schratt G. (2009). microRNAs at the synapse. Nat. Rev. Neurosci..

[68] Woldemichael B.T., Mansuy I.M. (2016). Micro-RNAs in cognition and cognitive disorders: Potential for novel biomarkers and therapeutics. Biochem. Pharmacol..

[69] Packer A.N., Xing Y., Harper S.Q., Jones L., Davidson B.L. (2008). The bifunctional microRNA miR-9/miR-9* regulates REST and CoREST and is downregulated in Huntington’s disease. J. Neurosci..

[70] Schiffer D., Caldera V., Mellai M., Conforti P., Cattaneo E., Zuccato C. (2014). Repressor element-1 silencing transcription factor (REST) is present in human control and Huntington’s disease neurones. Neuropathol. Appl. Neurobiol..

[71] Tsai M.C., Manor O., Wan Y., Mosammaparast N., Wang J.K., Lan F., Shi Y., Segal E., Chang H.Y. (2010). Long noncoding RNA as modular scaffold of histone modification complexes. Science.

[72] O’Brien R.J., Wong P.C. (2011). Amyloid precursor protein processing and Alzheimer’s disease. Annu. Rev. Neurosci..

[73] DeTure M.A., Dickson D.W. (2019). The neuropathological diagnosis of Alzheimer’s disease. Mol. Neurodegener..

[74] Murgas P., Godoy B., von Bernhardi R. (2012). Abeta potentiates inflammatory activation of glial cells induced by scavenger receptor ligands and inflammatory mediators in culture. Neurotox. Res..

[75] Mandelkow E.M., Mandelkow E. (2012). Biochemistry and cell biology of tau protein in neurofibrillary degeneration. Cold Spring Harb. Perspect. Med..

[76] Hardy J., Duff K., Hardy K.G., Perez-Tur J., Hutton M. (1998). Genetic dissection of Alzheimer’s disease and related dementias: Amyloid and its relationship to tau. Nat. Neurosci..

[77] Masters C.L., Simms G., Weinman N.A., Multhaup G., McDonald B.L., Beyreuther K. (1985). Amyloid plaque core protein in Alzheimer disease and Down syndrome. Proc. Natl. Acad. Sci. USA.

[78] Glass C.K., Saijo K., Winner B., Marchetto M.C., Gage F.H. (2010). Mechanisms underlying inflammation in neurodegeneration. Cell.

[79] Allen N.J., Eroglu C. (2017). Cell Biology of Astrocyte-Synapse Interactions. Neuron.

[80] Verkhratsky A., Nedergaard M. (2018). Physiology of Astroglia. Physiol. Rev..

[81] Batiuk M.Y., Martirosyan A., Wahis J., de Vin F., Marneffe C., Kusserow C., Koeppen J., Viana J.F., Oliveira J.F., Voet T. (2020). Identification of region-specific astrocyte subtypes at single cell resolution. Nat. Commun..

[82] Endo F., Kasai A., Soto J.S., Yu X., Qu Z., Hashimoto H., Gradinaru V., Kawaguchi R., Khakh B.S. (2022). Molecular basis of astrocyte diversity and morphology across the CNS in health and disease. Science.

[83] Swardfager W., Lanctot K., Rothenburg L., Wong A., Cappell J., Herrmann N. (2010). A meta-analysis of cytokines in Alzheimer’s disease. Biol. Psychiatry.

[84] El Kadmiri N., Said N., Slassi I., El Moutawakil B., Nadifi S. (2018). Biomarkers for Alzheimer Disease: Classical and Novel Candidates’ Review. Neuroscience.

[85] Edison P., Brooks D.J. (2018). Role of Neuroinflammation in the Trajectory of Alzheimer’s Disease and in vivo Quantification Using PET. J. Alzheimers Dis..

[86] Beaino W., Janssen B., Kooij G., van der Pol S.M.A., van Het Hof B., van Horssen J., Windhorst A.D., de Vries H.E. (2017). Purinergic receptors P2Y12R and P2X7R: Potential targets for PET imaging of microglia phenotypes in multiple sclerosis. J. Neuroinflamm..

[87] Narayanaswami V., Dahl K., Bernard-Gauthier V., Josephson L., Cumming P., Vasdev N. (2018). Emerging PET Radiotracers and Targets for Imaging of Neuroinflammation in Neurodegenerative Diseases: Outlook Beyond TSPO. Mol. Imaging.

[88] Husain M.A., Laurent B., Plourde M. (2021). APOE and Alzheimer’s Disease: From Lipid Transport to Physiopathology and Therapeutics. Front. Neurosci..

[89] Mise A., Yoshino Y., Yamazaki K., Ozaki Y., Sao T., Yoshida T., Mori T., Mori Y., Ochi S., Iga J.I. (2017). TOMM40 and APOE Gene Expression and Cognitive Decline in Japanese Alzheimer’s Disease Subjects. J. Alzheimers Dis..

[90] Lee E.G., Tulloch J., Chen S., Leong L., Saxton A.D., Kraemer B., Darvas M., Keene C.D., Shutes-David A., Todd K. (2020). Redefining transcriptional regulation of the APOE gene and its association with Alzheimer’s disease. PLoS ONE.

[91] Shao Y., Shaw M., Todd K., Khrestian M., D’Aleo G., Barnard P.J., Zahratka J., Pillai J., Yu C.E., Keene C.D. (2018). DNA methylation of TOMM40-APOE-APOC2 in Alzheimer’s disease. J. Hum. Genet..

[92] Nagata T., Kobayashi N., Ishii J., Shinagawa S., Nakayama R., Shibata N., Kuerban B., Ohnuma T., Kondo K., Arai H. (2015). Association between DNA Methylation of the BDNF Promoter Region and Clinical Presentation in Alzheimer’s Disease. Dement. Geriatr. Cogn. Dis. Extra.

[93] Nicolia V., Ciraci V., Cavallaro R.A., Ferrer I., Scarpa S., Fuso A. (2017). GSK3beta 5′-flanking DNA Methylation and Expression in Alzheimer’s Disease Patients. Curr. Alzheimer. Res..

[94] Ozaki Y., Yoshino Y., Yamazaki K., Sao T., Mori Y., Ochi S., Yoshida T., Mori T., Iga J.I., Ueno S.I. (2017). DNA methylation changes at TREM2 intron 1 and TREM2 mRNA expression in patients with Alzheimer’s disease. J. Psychiatr. Res..

[95] Smith A.R., Smith R.G., Burrage J., Troakes C., Al-Sarraj S., Kalaria R.N., Sloan C., Robinson A.C., Mill J., Lunnon K. (2019). A cross-brain regions study of ANK1 DNA methylation in different neurodegenerative diseases. Neurobiol. Aging.

[96] Semick S.A., Bharadwaj R.A., Collado-Torres L., Tao R., Shin J.H., Deep-Soboslay A., Weiss J.R., Weinberger D.R., Hyde T.M., Kleinman J.E. (2019). Integrated DNA methylation and gene expression profiling across multiple brain regions implicate novel genes in Alzheimer’s disease. Acta Neuropathol..

[97] Villela D., Ramalho R.F., Silva A.R., Brentani H., Suemoto C.K., Pasqualucci C.A., Grinberg L.T., Krepischi A.C., Rosenberg C. (2016). Differential DNA Methylation of MicroRNA Genes in Temporal Cortex from Alzheimer’s Disease Individuals. Neural Plast..

[98] Watson C.T., Roussos P., Garg P., Ho D.J., Azam N., Katsel P.L., Haroutunian V., Sharp A.J. (2016). Genome-wide DNA methylation profiling in the superior temporal gyrus reveals epigenetic signatures associated with Alzheimer’s disease. Genome Med..

[99] Li P., Marshall L., Oh G., Jakubowski J.L., Groot D., He Y., Wang T., Petronis A., Labrie V. (2019). Epigenetic dysregulation of enhancers in neurons is associated with Alzheimer’s disease pathology and cognitive symptoms. Nat. Commun..

[100] Coppieters N., Dieriks B.V., Lill C., Faull R.L., Curtis M.A., Dragunow M. (2014). Global changes in DNA methylation and hydroxymethylation in Alzheimer’s disease human brain. Neurobiol. Aging.

[101] Fetahu I.S., Ma D., Rabidou K., Argueta C., Smith M., Liu H., Wu F., Shi Y.G. (2019). Epigenetic signatures of methylated DNA cytosine in Alzheimer’s disease. Sci. Adv..

[102] Hroudova J., Singh N., Fisar Z. (2014). Mitochondrial dysfunctions in neurodegenerative diseases: Relevance to Alzheimer’s disease. Biomed. Res. Int..

[103] Shock L.S., Thakkar P.V., Peterson E.J., Moran R.G., Taylor S.M. (2011). DNA methyltransferase 1, cytosine methylation, and cytosine hydroxymethylation in mammalian mitochondria. Proc. Natl. Acad. Sci. USA.

[104] Kang K.A., Hyun J.W. (2017). Oxidative Stress, Nrf2, and Epigenetic Modification Contribute to Anticancer Drug Resistance. Toxicol. Res..

[105] Blanch M., Mosquera J.L., Ansoleaga B., Ferrer I., Barrachina M. (2016). Altered Mitochondrial DNA Methylation Pattern in Alzheimer Disease-Related Pathology and in Parkinson Disease. Am. J. Pathol..

[106] Stoccoro A., Siciliano G., Migliore L., Coppede F. (2017). Decreased Methylation of the Mitochondrial D-Loop Region in Late-Onset Alzheimer’s Disease. J. Alzheimers Dis..

[107] Mastroeni D., Grover A., Delvaux E., Whiteside C., Coleman P.D., Rogers J. (2011). Epigenetic mechanisms in Alzheimer’s disease. Neurobiol. Aging.

[108] Frost B., Hemberg M., Lewis J., Feany M.B. (2014). Tau promotes neurodegeneration through global chromatin relaxation. Nat. Neurosci..

[109] Marzi S.J., Leung S.K., Ribarska T., Hannon E., Smith A.R., Pishva E., Poschmann J., Moore K., Troakes C., Al-Sarraj S. (2018). A histone acetylome-wide association study of Alzheimer’s disease identifies disease-associated H3K27ac differences in the entorhinal cortex. Nat. Neurosci..

[110] Klein H.U., McCabe C., Gjoneska E., Sullivan S.E., Kaskow B.J., Tang A., Smith R.V., Xu J., Pfenning A.R., Bernstein B.E. (2019). Epigenome-wide study uncovers large-scale changes in histone acetylation driven by tau pathology in aging and Alzheimer’s human brains. Nat. Neurosci..

[111] Berson A., Nativio R., Berger S.L., Bonini N.M. (2018). Epigenetic Regulation in Neurodegenerative Diseases. Trends Neurosci..

[112] Stoccoro A., Coppede F. (2018). Role of epigenetics in Alzheimer’s disease pathogenesis. Neurodegener. Dis. Manag..

[113] Liu X., Jiao B., Shen L. (2018). The Epigenetics of Alzheimer’s Disease: Factors and Therapeutic Implications. Front. Genet..

[114] Zhang L., Sheng S., Qin C. (2013). The role of HDAC6 in Alzheimer’s disease. J. Alzheimers Dis..

[115] Xu K., Dai X.L., Huang H.C., Jiang Z.F. (2011). Targeting HDACs: A promising therapy for Alzheimer’s disease. Oxid. Med. Cell. Longev..

[116] Lu X., Wang L., Yu C., Yu D., Yu G. (2015). Histone Acetylation Modifiers in the Pathogenesis of Alzheimer’s Disease. Front. Cell. Neurosci..

[117] Konsoula Z., Barile F.A. (2012). Epigenetic histone acetylation and deacetylation mechanisms in experimental models of neurodegenerative disorders. J. Pharmacol. Toxicol. Methods.

[118] Nikolac Perkovic M., Videtic Paska A., Konjevod M., Kouter K., Svob Strac D., Nedic Erjavec G., Pivac N. (2021). Epigenetics of Alzheimer’s Disease. Biomolecules.

[119] Fischer A. (2014). Targeting histone-modifications in Alzheimer’s disease. What is the evidence that this is a promising therapeutic avenue?. Neuropharmacology.

[120] Herrera-Espejo S., Santos-Zorrozua B., Alvarez-Gonzalez P., Lopez-Lopez E., Garcia-Orad A. (2019). A Systematic Review of MicroRNA Expression as Biomarker of Late-Onset Alzheimer’s Disease. Mol. Neurobiol..

[121] Holohan K.N., Lahiri D.K., Schneider B.P., Foroud T., Saykin A.J. (2012). Functional microRNAs in Alzheimer’s disease and cancer: Differential regulation of common mechanisms and pathways. Front. Genet..

[122] Chang F., Zhang L.H., Xu W.P., Jing P., Zhan P.Y. (2014). microRNA-9 attenuates amyloidbeta-induced synaptotoxicity by targeting calcium/calmodulin-dependent protein kinase kinase 2. Mol. Med. Rep..

[123] Miya Shaik M., Tamargo I.A., Abubakar M.B., Kamal M.A., Greig N.H., Gan S.H. (2018). The Role of microRNAs in Alzheimer’s Disease and Their Therapeutic Potentials. Genes.

[124] Lukiw W.J., Zhao Y., Cui J.G. (2008). An NF-kappaB-sensitive micro RNA-146a-mediated inflammatory circuit in Alzheimer disease and in stressed human brain cells. J. Biol. Chem..

[125] Shioya M., Obayashi S., Tabunoki H., Arima K., Saito Y., Ishida T., Satoh J. (2010). Aberrant microRNA expression in the brains of neurodegenerative diseases: miR-29a decreased in Alzheimer disease brains targets neurone navigator 3. Neuropathol. Appl. Neurobiol..

[126] Wang W.X., Rajeev B.W., Stromberg A.J., Ren N., Tang G., Huang Q., Rigoutsos I., Nelson P.T. (2008). The expression of microRNA miR-107 decreases early in Alzheimer’s disease and may accelerate disease progression through regulation of beta-site amyloid precursor protein-cleaving enzyme 1. J. Neurosci..

[127] Kole A.J., Swahari V., Hammond S.M., Deshmukh M. (2011). miR-29b is activated during neuronal maturation and targets BH3-only genes to restrict apoptosis. Genes Dev..

[128] Rohn T.T., Vyas V., Hernandez-Estrada T., Nichol K.E., Christie L.A., Head E. (2008). Lack of pathology in a triple transgenic mouse model of Alzheimer’s disease after overexpression of the anti-apoptotic protein Bcl-2. J. Neurosci..

[129] Banzhaf-Strathmann J., Benito E., May S., Arzberger T., Tahirovic S., Kretzschmar H., Fischer A., Edbauer D. (2014). MicroRNA-125b induces tau hyperphosphorylation and cognitive deficits in Alzheimer’s disease. EMBO J..

[130] Smith P.Y., Hernandez-Rapp J., Jolivette F., Lecours C., Bisht K., Goupil C., Dorval V., Parsi S., Morin F., Planel E. (2015). miR-132/212 deficiency impairs tau metabolism and promotes pathological aggregation in vivo. Hum. Mol. Genet..

[131] Geekiyanage H., Chan C. (2011). MicroRNA-137/181c regulates serine palmitoyltransferase and in turn amyloid beta, novel targets in sporadic Alzheimer’s disease. J. Neurosci..

[132] Li J.J., Wang B., Kodali M.C., Chen C., Kim E., Patters B.J., Lan L., Kumar S., Wang X., Yue J. (2018). In vivo evidence for the contribution of peripheral circulating inflammatory exosomes to neuroinflammation. J. Neuroinflamm..

[133] Jankovic J., Tan E.K. (2020). Parkinson’s disease: Etiopathogenesis and treatment. J. Neurol. Neurosurg. Psychiatry.

[134] Ascherio A., Schwarzschild M.A. (2016). The epidemiology of Parkinson’s disease: Risk factors and prevention. Lancet Neurol..

[135] Hawkes C.H., Del Tredici K., Braak H. (2007). Parkinson’s disease: A dual-hit hypothesis. Neuropathol. Appl. Neurobiol..

[136] Takahashi S., Mashima K. (2022). Neuroprotection and Disease Modification by Astrocytes and Microglia in Parkinson Disease. Antioxidants.

[137] Jewell S., Herath A.M., Gordon R. (2022). Inflammasome Activation in Parkinson’s Disease. J. Parkinsons Dis..

[138] Tang Y., Li T., Li J., Yang J., Liu H., Zhang X.J., Le W. (2014). Jmjd3 is essential for the epigenetic modulation of microglia phenotypes in the immune pathogenesis of Parkinson’s disease. Cell Death Differ..

[139] Long-Smith C.M., Collins L., Toulouse A., Sullivan A.M., Nolan Y.M. (2010). Interleukin-1beta contributes to dopaminergic neuronal death induced by lipopolysaccharide-stimulated rat glia in vitro. J. Neuroimmunol..

[140] Na S.J., DiLella A.G., Lis E.V., Jones K., Levine D.M., Stone D.J., Hess J.F. (2010). Molecular profiling of a 6-hydroxydopamine model of Parkinson’s disease. Neurochem. Res..

[141] Tieu K. (2011). A guide to neurotoxic animal models of Parkinson’s disease. Cold Spring Harb. Perspect. Med..

[142] Tapias V., Hu X., Luk K.C., Sanders L.H., Lee V.M., Greenamyre J.T. (2017). Synthetic alpha-synuclein fibrils cause mitochondrial impairment and selective dopamine neurodegeneration in part via iNOS-mediated nitric oxide production. Cell Mol. Life Sci..

[143] Theodore S., Cao S., McLean P.J., Standaert D.G. (2008). Targeted overexpression of human alpha-synuclein triggers microglial activation and an adaptive immune response in a mouse model of Parkinson disease. J. Neuropathol. Exp. Neurol..

[144] Booth H.D.E., Hirst W.D., Wade-Martins R. (2017). The Role of Astrocyte Dysfunction in Parkinson’s Disease Pathogenesis. Trends Neurosci..

[145] Lee H.J., Suk J.E., Patrick C., Bae E.J., Cho J.H., Rho S., Hwang D., Masliah E., Lee S.J. (2010). Direct transfer of alpha-synuclein from neuron to astroglia causes inflammatory responses in synucleinopathies. J. Biol. Chem..

[146] Pajares M.I., Rojo A., Manda G., Bosca L., Cuadrado A. (2020). Inflammation in Parkinson’s Disease: Mechanisms and Therapeutic Implications. Cells.

[147] Hirsch E.C., Breidert T., Rousselet E., Hunot S., Hartmann A., Michel P.P. (2003). The role of glial reaction and inflammation in Parkinson’s disease. Ann. N. Y. Acad. Sci..

[148] Guhathakurta S., Bok E., Evangelista B.A., Kim Y.S. (2017). Deregulation of alpha-synuclein in Parkinson’s disease: Insight from epigenetic structure and transcriptional regulation of SNCA. Prog. Neurobiol..

[149] Desplats P., Spencer B., Coffee E., Patel P., Michael S., Patrick C., Adame A., Rockenstein E., Masliah E. (2011). Alpha-synuclein sequesters Dnmt1 from the nucleus: A novel mechanism for epigenetic alterations in Lewy body diseases. J. Biol. Chem..

[150] Chen S., Bellew C., Yao X., Stefkova J., Dipp S., Saifudeen Z., Bachvarov D., El-Dahr S.S. (2011). Histone deacetylase (HDAC) activity is critical for embryonic kidney gene expression, growth, and differentiation. J. Biol. Chem..

[151] van Heesbeen H.J., Smidt M.P. (2019). Entanglement of Genetics and Epigenetics in Parkinson’s Disease. Front. Neurosci..

[152] Mittal S., Bjornevik K., Im D.S., Flierl A., Dong X., Locascio J.J., Abo K.M., Long E., Jin M., Xu B. (2017). beta2-Adrenoreceptor is a regulator of the alpha-synuclein gene driving risk of Parkinson’s disease. Science.

[153] Toker L., Tran G.T., Sundaresan J., Tysnes O.B., Alves G., Haugarvoll K., Nido G.S., Dolle C., Tzoulis C. (2021). Genome-wide histone acetylation analysis reveals altered transcriptional regulation in the Parkinson’s disease brain. Mol. Neurodegener..

[154] Jing H., Lin H. (2015). Sirtuins in epigenetic regulation. Chem. Rev..

[155] Chio A., Logroscino G., Hardiman O., Swingler R., Mitchell D., Beghi E., Traynor B.G., Eurals C. (2009). Prognostic factors in ALS: A critical review. Amyotroph. Lateral Scler..

[156] Lomen-Hoerth C., Anderson T., Miller B. (2002). The overlap of amyotrophic lateral sclerosis and frontotemporal dementia. Neurology.

[157] Ince P.G., Highley J.R., Kirby J., Wharton S.B., Takahashi H., Strong M.J., Shaw P.J. (2011). Molecular pathology and genetic advances in amyotrophic lateral sclerosis: An emerging molecular pathway and the significance of glial pathology. Acta Neuropathol..

[158] Tarr I.S., McCann E.P., Benyamin B., Peters T.J., Twine N.A., Zhang K.Y., Zhao Q., Zhang Z.H., Rowe D.B., Nicholson G.A. (2019). Monozygotic twins and triplets discordant for amyotrophic lateral sclerosis display differential methylation and gene expression. Sci. Rep..

[159] Vicario N., Spitale F.M., Tibullo D., Giallongo C., Amorini A.M., Scandura G., Spoto G., Saab M.W., D’Aprile S., Alberghina C. (2021). Clobetasol promotes neuromuscular plasticity in mice after motoneuronal loss via sonic hedgehog signaling, immunomodulation and metabolic rebalancing. Cell Death Dis..

[160] Liu E., Karpf L., Bohl D. (2021). Neuroinflammation in Amyotrophic Lateral Sclerosis and Frontotemporal Dementia and the Interest of Induced Pluripotent Stem Cells to Study Immune Cells Interactions With Neurons. Front. Mol. Neurosci..

[161] Gandelman M., Peluffo H., Beckman J.S., Cassina P., Barbeito L. (2010). Extracellular ATP and the P2X7 receptor in astrocyte-mediated motor neuron death: Implications for amyotrophic lateral sclerosis. J. Neuroinflamm..

[162] Apolloni S., Amadio S., Parisi C., Matteucci A., Potenza R.L., Armida M., Popoli P., D’Ambrosi N., Volonte C. (2014). Spinal cord pathology is ameliorated by P2X7 antagonism in a SOD1-mutant mouse model of amyotrophic lateral sclerosis. Dis. Model Mech..

[163] Bartlett R., Sluyter V., Watson D., Sluyter R., Yerbury J.J. (2017). P2X7 antagonism using Brilliant Blue G reduces body weight loss and prolongs survival in female SOD1(G93A) amyotrophic lateral sclerosis mice. PeerJ.

[164] Yamanaka K., Komine O. (2018). The multi-dimensional roles of astrocytes in ALS. Neurosci. Res..

[165] Coppede F., Stoccoro A. (2019). Mitoepigenetics and Neurodegenerative Diseases. Front. Endocrinol..

[166] Stoccoro A., Mosca L., Carnicelli V., Cavallari U., Lunetta C., Marocchi A., Migliore L., Coppede F. (2018). Mitochondrial DNA copy number and D-loop region methylation in carriers of amyotrophic lateral sclerosis gene mutations. Epigenomics.

[167] Chevin M., Sebire G. (2021). Necroptosis in ALS: A hot topic in-progress. Cell Death Discov..

[168] Brown R.H., Al-Chalabi A. (2017). Amyotrophic Lateral Sclerosis. N. Engl. J. Med..

[169] Hardiman O., Al-Chalabi A., Chio A., Corr E.M., Logroscino G., Robberecht W., Shaw P.J., Simmons Z., van den Berg L.H. (2017). Amyotrophic lateral sclerosis. Nat. Rev. Dis. Primers.

[170] Lian L., Liu M., Cui L., Guan Y., Liu T., Cui B., Zhang K., Tai H., Shen D. (2019). Environmental risk factors and amyotrophic lateral sclerosis (ALS): A case-control study of ALS in China. J. Clin. Neurosci..

[171] Dickerson A.S., Hansen J., Thompson S., Gredal O., Weisskopf M.G. (2020). A mixtures approach to solvent exposures and amyotrophic lateral sclerosis: A population-based study in Denmark. Eur. J. Epidemiol..

[172] Bellavia A., Dickerson A.S., Rotem R.S., Hansen J., Gredal O., Weisskopf M.G. (2021). Joint and interactive effects between health comorbidities and environmental exposures in predicting amyotrophic lateral sclerosis. Int. J. Hyg. Environ. Health.

[173] Figueroa-Romero C., Hur J., Bender D.E., Delaney C.E., Cataldo M.D., Smith A.L., Yung R., Ruden D.M., Callaghan B.C., Feldman E.L. (2012). Identification of epigenetically altered genes in sporadic amyotrophic lateral sclerosis. PLoS ONE.

[174] Cai Z., Jia X., Liu M., Yang X., Cui L. (2022). Epigenome-wide DNA methylation study of whole blood in patients with sporadic amyotrophic lateral sclerosis. Chin. Med. J..

[175] Oates N., Pamphlett R. (2007). An epigenetic analysis of SOD1 and VEGF in ALS. Amyotroph. Lateral Scler..

[176] Rothstein J.D., Van Kammen M., Levey A.I., Martin L.J., Kuncl R.W. (1995). Selective loss of glial glutamate transporter GLT-1 in amyotrophic lateral sclerosis. Ann. Neurol..

[177] Stoccoro A., Smith A.R., Mosca L., Marocchi A., Gerardi F., Lunetta C., Cereda C., Gagliardi S., Lunnon K., Migliore L. (2020). Reduced mitochondrial D-loop methylation levels in sporadic amyotrophic lateral sclerosis. Clin. Epigenetics.

[178] Orton S.M., Herrera B.M., Yee I.M., Valdar W., Ramagopalan S.V., Sadovnick A.D., Ebers G.C., Canadian Collaborative Study Group (2006). Sex ratio of multiple sclerosis in Canada: A longitudinal study. Lancet Neurol..

[179] Compston A., Coles A. (2008). Multiple sclerosis. Lancet.

[180] Gale C.R., Martyn C.N. (1995). Migrant studies in multiple sclerosis. Prog. Neurobiol..

[181] Hedstrom A.K., Sundqvist E., Baarnhielm M., Nordin N., Hillert J., Kockum I., Olsson T., Alfredsson L. (2011). Smoking and two human leukocyte antigen genes interact to increase the risk for multiple sclerosis. Brain.

[182] Ferguson B., Matyszak M.K., Esiri M.M., Perry V.H. (1997). Axonal damage in acute multiple sclerosis lesions. Brain.

[183] Trapp B.D., Peterson J., Ransohoff R.M., Rudick R., Mork S., Bo L. (1998). Axonal transection in the lesions of multiple sclerosis. N. Engl. J. Med..

[184] Chandran P.K., Kuttan R. (2008). Effect of Calendula officinalis Flower Extract on Acute Phase Proteins, Antioxidant Defense Mechanism and Granuloma Formation During Thermal Burns. J. Clin. Biochem. Nutr..

[185] Cossburn M., Tackley G., Baker K., Ingram G., Burtonwood M., Malik G., Pickersgill T., te Water Naude J., Robertson N. (2012). The prevalence of neuromyelitis optica in South East Wales. Eur. J. Neurol..

[186] Kalincik T., Buzzard K., Jokubaitis V., Trojano M., Duquette P., Izquierdo G., Girard M., Lugaresi A., Grammond P., Grand’Maison F. (2014). Risk of relapse phenotype recurrence in multiple sclerosis. Mult. Scler..

[187] Confavreux C., Vukusic S. (2006). Natural history of multiple sclerosis: A unifying concept. Brain.

[188] Tutuncu M., Tang J., Zeid N.A., Kale N., Crusan D.J., Atkinson E.J., Siva A., Pittock S.J., Pirko I., Keegan B.M. (2013). Onset of progressive phase is an age-dependent clinical milestone in multiple sclerosis. Mult. Scler..

[189] Penna G., Roncari A., Amuchastegui S., Daniel K.C., Berti E., Colonna M., Adorini L. (2005). Expression of the inhibitory receptor ILT3 on dendritic cells is dispensable for induction of CD4+Foxp3+ regulatory T cells by 1,25-dihydroxyvitamin D3. Blood.

[190] Ma Q., Oksenberg J.R., Didonna A. (2022). Epigenetic control of ataxin-1 in multiple sclerosis. Ann. Clin. Transl. Neurol..

[191] Didonna A., Canto Puig E., Ma Q., Matsunaga A., Ho B., Caillier S.J., Shams H., Lee N., Hauser S.L., Tan Q. (2020). Ataxin-1 regulates B cell function and the severity of autoimmune experimental encephalomyelitis. Proc. Natl. Acad. Sci. USA.

[192] Lee Y., Samaco R.C., Gatchel J.R., Thaller C., Orr H.T., Zoghbi H.Y. (2008). miR-19, miR-101 and miR-130 co-regulate ATXN1 levels to potentially modulate SCA1 pathogenesis. Nat. Neurosci..

[193] Gennarino V.A., Singh R.K., White J.J., De Maio A., Han K., Kim J.Y., Jafar-Nejad P., di Ronza A., Kang H., Sayegh L.S. (2015). Pumilio1 haploinsufficiency leads to SCA1-like neurodegeneration by increasing wild-type Ataxin1 levels. Cell.

[194] Lowe K., Wu E., Wang N., Hoerster G., Hastings C., Cho M.J., Scelonge C., Lenderts B., Chamberlin M., Cushatt J. (2016). Morphogenic Regulators Baby boom and Wuschel Improve Monocot Transformation. Plant Cell.

[195] Horvath S., Raj K. (2018). DNA methylation-based biomarkers and the epigenetic clock theory of ageing. Nat. Rev. Genet..

[196] Merrill J.E., Scolding N.J. (1999). Mechanisms of damage to myelin and oligodendrocytes and their relevance to disease. Neuropathol. Appl. Neurobiol..

[197] Miyachi H., Konishi T., Kumazawa R., Matsui H., Shimizu S., Fushimi K., Matsue H., Yasunaga H. (2022). Treatments and outcomes of generalized pustular psoriasis: A cohort of 1516 patients in a nationwide inpatient database in Japan. J. Am. Acad. Dermatol..

[198] Luo C., Keown C.L., Kurihara L., Zhou J., He Y., Li J., Castanon R., Lucero J., Nery J.R., Sandoval J.P. (2017). Single-cell methylomes identify neuronal subtypes and regulatory elements in mammalian cortex. Science.

[199] Ponath G., Park C., Pitt D. (2018). The Role of Astrocytes in Multiple Sclerosis. Front. Immunol..

[200] Kular L., Klose D., Urdanoz-Casado A., Ewing E., Planell N., Gomez-Cabrero D., Needhamsen M., Jagodic M. (2022). Epigenetic clock indicates accelerated aging in glial cells of progressive multiple sclerosis patients. Front. Aging Neurosci..

